# Adsorption and Structuration of PEG Thin Films: Influence of the Substrate Chemistry

**DOI:** 10.3390/polym16091244

**Published:** 2024-04-29

**Authors:** Maurice Brogly, Sophie Bistac, Diane Bindel

**Affiliations:** Laboratoire de Photochimie et d’Ingénierie Macromoléculaires, Université de Haute Alsace, 3b Rue Alfred Werner, 68093 Mulhouse, France

**Keywords:** polyethylene glycol (PEG), thin film, surface chemistry, PM-IRRAS spectrometry, structuration

## Abstract

This study investigates polyethylene glycol (PEG) homopolymer thin film adsorption on gold surfaces of controlled surface chemistry. The conformational states of physisorbed PEG are analyzed through polarization modulation infrared reflection–absorption spectrometry (PM-IRRAS). The PM-IRRAS principle is based on specific optical selection rules allowing the detection of surface-specific FTIR response of thin polymer films on the basis of differential reflectivity at the polymer/substrate interface for p- and s-polarized light. The intensification of the electric field generated at the PEG/substrate interface for p-polarized IR light in comparison with s-polarized light permits the analysis of PEG chain anisotropy and conformational changes induced by the adsorption. Results showed that PEG adsorbs on model substrates having a rather hydrophilic character in a way that the PEG chains spread parallel to the surface. In the case of a very hydrophilic substrate, the adsorbed PEG chains are in a stable thermodynamic state which allows them to arrange and crystallize as stacked crystalline lamellae after adsorption. The surface topography and morphology of the PEG thin films were also investigated by atomic force microscopy (AFM). While in the bulk state, PEG crystallizes in the form of large spherulites; on substrates whose adsorption is favored by surface chemistry, PEG crystallizes in the form of stacked lamellae with a thickness equal to 20 nm. Conversely, on a hydrophobic substrate, the PEG chains do not crystallize and adsorption occurs in the statistical coil state.

## 1. Introduction

Polyethylene glycol (PEG) is a biocompatible semi-crystalline polymer with hydrophilic behavior that can be synthesized by anionic, cationic or opening polymerization of an ethylene glycol monomer. PEG is used in the composition of a large number of industrial products, such as binders and bases in cosmetic formulations, plasticizers in adhesives, detergents in household products due to their solubility in water and low toxicity, or as a co-solvent of water, as it reduces solution polarity to favor the solubility of organic molecules. PEG is thus a polymer of great interest for various applications, particularly in the field of biotechnology. Indeed, being biocompatible and non-toxic, PEG does not damage proteins or biological cells. Its hydrophilic character allows it to repel other polymers in aqueous solution [1], which makes it a very good candidate as an encapsulating agent [2]. This property is exploited by Pean et al. [3] to stabilize a protein during the encapsulation process by using PEG with a 400 g.mol^−1^ molar mass. The hydrophilicity of PEG also allowed Albertsson [4] to discover that a mixture of PEG and dextran forms a two-phase polymer system, allowing the purification of biological compounds [4]. In addition to not damaging biological cells, PEG can interact with cell membranes to trigger cell fusion, which is a key process in the field of biotechnology [5,6]. PEGs with molar masses varying between 200 g.mol^−1^ and 20,000 g.mol^−1^ allow access to even more varied properties in the biomedical field when they bind covalently with proteins. Buchowski et al. showed that attachment by covalent bonding of PEG to proteins increases the lifetime of biological serums [7]. Mori et al. demonstrated that the attachment by covalent bonding of PEG to a surface considerably delays the adsorption of proteins on these surfaces [8]. Molecular characterization is therefore crucial for understanding, predicting and controlling PEG surface characteristics. IR spectroscopy is a specialized analytical method for the detection of conformational variations based on the advantage that bands related to repeating unit sequences in crystalline, amorphous and intermediate phases are observed independently in the IR spectra [9]. The objective of this study is to investigate the adsorption and organization of crystalline PEG polymer in thin films. In that way, polarization modulation infrared reflection–absorption spectrometry (PM-IRRAS) is used to access the PEG chain conformational states when physisorbed as thin films on a substrate. The measure of surface PM-IRRAS spectra is possible due to the differential reflectivity of p- and s-polarized light [10,11]. The magnification of the electric field at the PEG/substrate interface allows inferring PEG adsorption, structuration and conformational changes induced by PEG/substrate interactions. The gold-coated glass substrate was chosen for its high reflectance and low roughness, as well as its chemical and inertness properties to provide insight into the effects of confinement on adsorption and PEG polymer chain structure. The advantage of gold is also the possibility to modify its surface chemistry by thiol grafting. Therefore, the influence of the chemistry of the substrate (hydrophilic vs. hydrophobic) on the adsorption and structuration of PEG thin films will be studied. In addition to spectroscopic analysis, the surface topology of the PEG thin films and their ability to crystallize at solid interfaces were examined by atomic force microscopy (AFM).

## 2. Materials and Methods

### 2.1. Materials

**Figure 1:**
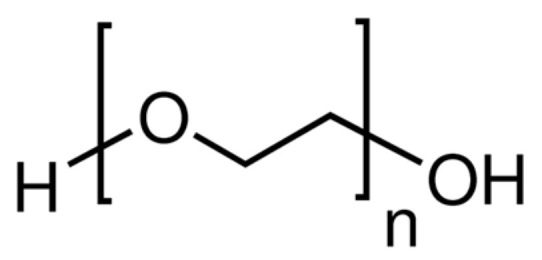
Formula of polyethylene glycol (PEG).

Polyethylene glycol with Mn = 2050 g.mol^−1^ (Sigma Aldrich, St. Louis, MO, USA) and conditioned as flakes was used as received for the bulk characterization by infrared attenuated total reflection (ATR) spectroscopy. The formula of this polymer is given in Figure 1. Several studies characterized the high crystallinity of PEG varying from 85.7% to 87.2% for Mn = 1000 g.mol^−1^ and Mn = 3400 g.mol^−1^, respectively [12]. PEG with Mn = 2000 g.mol^−1^ has been studied by Li et al. [13], revealing a degree of crystallinity equal to 94%.

For the PM-IRRAS experiments, PEG was dissolved in THF (5 g.L^−1^) and spin-coated (1000 rpm for 30 s) on different substrates. The thin films were dried in ambient air. The thicknesses of the films were around 20 nm. In order to better understand the effect of adsorption on the chain organization at the solid surface, PEG was spin-coated from solutions on gold-coated glass substrates. This substrate offers two main advantages for the study: a highly reflective surface for the PM-IRRAS spectroscopic analysis and also a weak surface roughness for atomic force microscopy (AFM) analysis. Gold-coated substrates were prepared using glass slides, firstly cleaned by ultrasound for 5 min in a distilled water bath, then rinsed with acetone and ethanol. They were finally dried in an oven for 10 min at 80 °C. In order to ensure that the gold layer remains on the glass slide during chemical grafting, it is necessary to use a coupling agent between the glass slide and the gold layer. The surface was activated (surface enrichment with silanol groups) by immersion of the cleaned glass slides in a piranha solution (70% H_2_SO_4_ + 30% H_2_O_2_) at 50 °C for 15 min. A solution of 3-mercaptotriethoxysilane (ABCR) dissolved in toluene was used as a coupling agent between the glass slide and the gold layer. Quickly after rinsing with toluene a 50 nm thin layer of gold was deposited using a Cressington Serie 108 metallizer (Cressington Scientific Instruments, Watford, UK) under argon.

**Figure 2:**
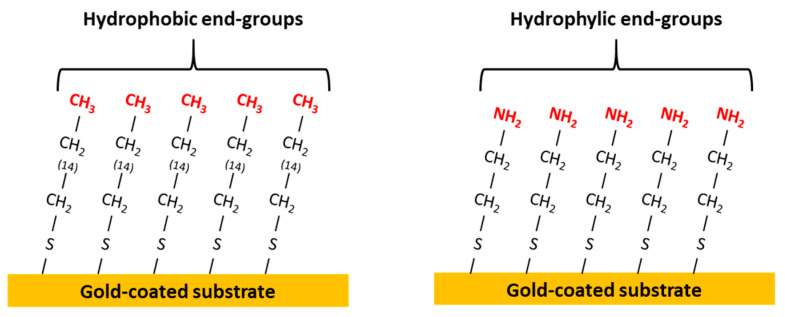
Hydrophobic (Au-CH_3_) and hydrophilic (Au-NH_2_) substrates obtained by chemical grafting of gold-coated glass substrates.

The influence of the substrate chemistry was studied with the development of hydrophobic and hydrophilic substrates. The thiol molecules used for the gold-coated substrate functionalization are the following: hexadecanethiol (Sigma Aldrich) for the hydrophobic substrate (end-group CH_3_) and cysteamine (Sigma Aldrich) for the hydrophilic substrate (end-group NH_2_). Gold-coated glass substrates were immersed at room temperature in graft solutions of thiols at a concentration of 3 mmol.L^−1^ dissolved in toluene for hexadecanethiol and a water/ethanol mixture for cysteamine. The immersion time in the grafting solution was fixed at 30 min for the hydrophilic substrate and 18 h for the hydrophobic substrate. After the adsorption procedure, the substrates were washed with toluene or ethanol to remove ungrafted thiol molecules and then dried. The chemically grafted gold substrates are schematically represented in Figure 2.

The complete covering of the grafted surface with thiols and also the wettability of the different substrates used in this study have been characterized by water contact angle measurements. Water droplets have a contact angle of 60° on the Au substrate, indicating partial wetting. The contact angle is reduced to 21° on the Au-NH_2_ substrate, which can be considered a hydrophilic substrate. Water droplets show a contact angle of 109° on the Au-CH_3_ substrate, which can be considered a hydrophobic substrate. These values are in agreement with the literature [14].

### 2.2. Methods

#### 2.2.1. Infrared Spectroscopy

- Attenuated Total Reflection (ATR)

Bulk PEG homopolymer was analyzed by single-reflection attenuated total reflection (ATR) infrared spectroscopy. Measurements were performed directly on PEG pellets with a Bruker Vertex 70 FTIR spectrometer (Bruker France SAS, Wissembourg, France) coupled to a Bruker Platinum ATR module mounted on a universal QuickLock device in the sample compartment. The ATR module is equipped with a diamond single-reflection crystal. The FTIR spectrum of PEG homopolymer was acquired from 4000 to 400 cm^−1^. The number of scans was fixed at 50 with a resolution of 4 cm^−1^. The reproducibility of the results was systematically checked. Spectral data were processed using the Bruker software system Opus 7.5.

- Polarization-Modulation Infrared Reflection-Absorption Spectroscopy (PM-IRRAS)

**Figure 3:**
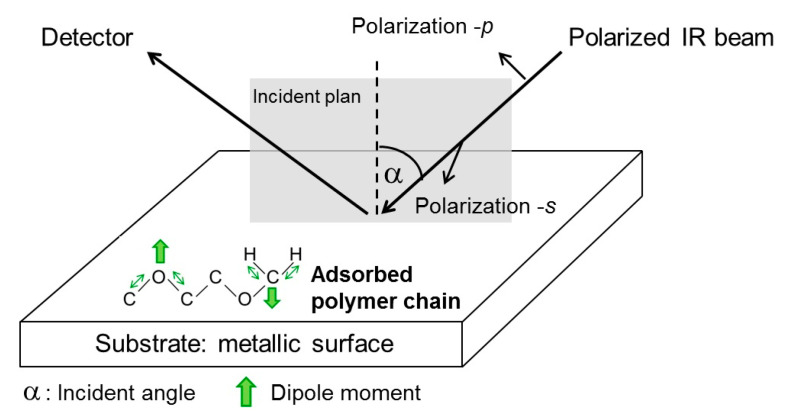
Scheme of the PM-IRRAS principle.

Since the amount of polymer deposited on the metal substrate is very small, it is very difficult to characterize the interface using conventional FTIR spectroscopy. PEG thin films on Au, Au-CH_3_ and Au-NH_2_ substrates were characterized by PM-IRRAS. This method detects an infrared beam that is polarized, doubly modulated, and then reflected from a surface, as shown in Figure 3. The difference in reflectivity of the two polarization components (s and p) is characteristic of the adsorption of the surface layers [15,16]. Thanks to the special selection rules caused by the polarization modulation and after mathematical demodulation of the signals, the measurement sensitivity is increased, and the signal noise is reduced [17], allowing molecular layer analysis of thicknesses in the nm range.

In addition to the high sensitivity and noise reduction, the selection rules at the reflection point of the IR waves imply a relation between the change in dipole moment direction for a given mode of vibration of a bond and the intensity of the corresponding PM-IRRAS absorption band. This technique is well known for providing access to the orientation of polymer chains adsorbed on a reflective substrate as thin films [18,19]. Indeed, when the dipole moment of a bond is oriented perpendicularly to the surface, the corresponding PM-IRRAS band is intensified. On the contrary, when the dipole moment is oriented parallel to the surface, the corresponding PM-IRRAS band is off. PM-IRRAS experiments were performed using a Bruker Vertex 70 FTIR spectrometer (Bruker France SAS, Wissembourg, France) equipped with a Bruker PMA 50 external optical rack for reflectance–absorption analysis. The module includes a photoelastic modulator (Hinds Instruments PM 100 (Hinds Instruments, Hillsboro, OR, USA)) with a 50 kHz ZnSe optical head, an MCT detector, and an SRS 830 lock-in amplifier (Stanford Research Systems, Sunnyvale, CA, USA) for synchronous demodulation of the signal. PM-IRRAS spectra of PEG thin films were recorded in the range of 4000–400 cm^−1^. The conditions for acquiring spectra were fixed at: 1000 scans and a resolution of 4 cm^−1^. All substrates were placed in a vertical configuration with an IR beam incidence angle of 80°, close to the grazing incidence angle. The reproducibility of the results was systematically checked. Spectral data were analyzed using the Bruker Opus 7.5 software package.

- Infrared Spectra Treatments

Several treatments were applied to both ATR and PM-IRRAS spectra in order to obtain the optimal spectral quality for the interpretation of the results. Diffusion, chromatic distortion and light diffraction are phenomena occurring when the infrared beam goes through the polymer sample in the bulk state or as thin films. A part of the light is thus deviated and does not reach the detector anymore, leading to an inclination of the baseline of the infrared spectra that is more or less important. A baseline correction was systematically applied to spectra. Unambiguous identification of the precise wavenumbers of characteristic IR vibration bands on each spectrum needs application of a second derivative mathematical treatment to the initial spectrum. The OPUS 7.5 software was used in that way. This function improves the resolution of the IR vibration bands, leading to more precise information in terms of band contribution and position.

#### 2.2.2. Atomic Force Microscopy (AFM)

AFM images of PEG thin films adsorbed on Au, Au-CH_3_ and Au-NH_2_ were obtained using a Bruker Dimension Edge AFM coupled to the Nanodrive 8.1 controller. AFM tips were supplied by Veeco Instruments, Plainview, NY, USA. All experiments were performed in contact mode. Tips are made of silicon nitride (Si_3_N_4_) with a gold reflective coating on the back side. The cantilever geometry is triangular, the spring constant is 0.35 N/m, allowing high sensitivity, and the tip radius is around 2 nm, allowing high vertical and lateral resolution. Scan sizes were fixed at 90 µm × 90 µm and 10 µm × 10 µm, and the scan frequency (lines/s) was fixed at 1 Hz. The AFM raw signal was recorded in deflection mode. The reproducibility of the results was systematically checked.

## 3. Results 

### 3.1. PEG Bulk Characterization Investigated by ATR

In order to better understand how semi-crystalline PEG is organized when adsorbed on a substrate, the IR spectral fingerprint of bulk PEG is firstly investigated. The FTIR attenuated total reflection (ATR) was performed on PEG pellets to assign the IR absorption bands observed in the spectrum to the corresponding bonds, functional groups and vibration modes. The coordinates of normal vibrations used for peak assignments are the following:

ν_a_: Antisymmetric stretching mode;

ν_s_: Symmetric stretching mode;

δ: Scissoring bending mode (in plane);

γ_r_: Rocking bending mode (in plane);

γ_w_: Wagging bending mode (out of plane);

γ_t_: Twisting (or torsion) bending mode (out of plane).

**Figure 4:**
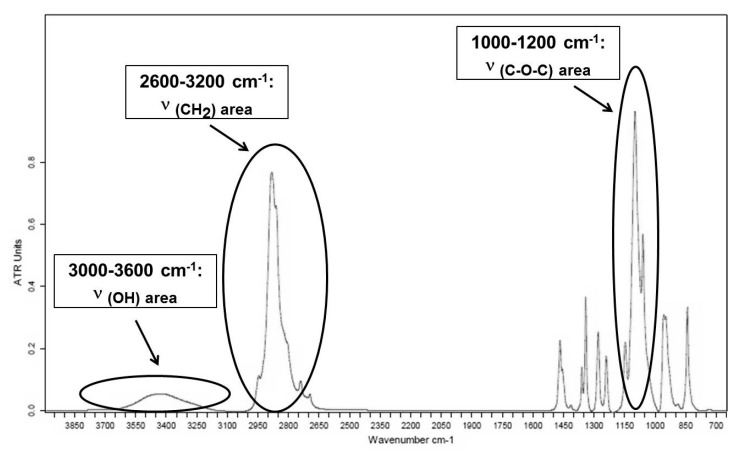
Infrared spectrum (ATR) of PEG in the 4000 to 700 cm^−1^ region.

**Figure 5:**
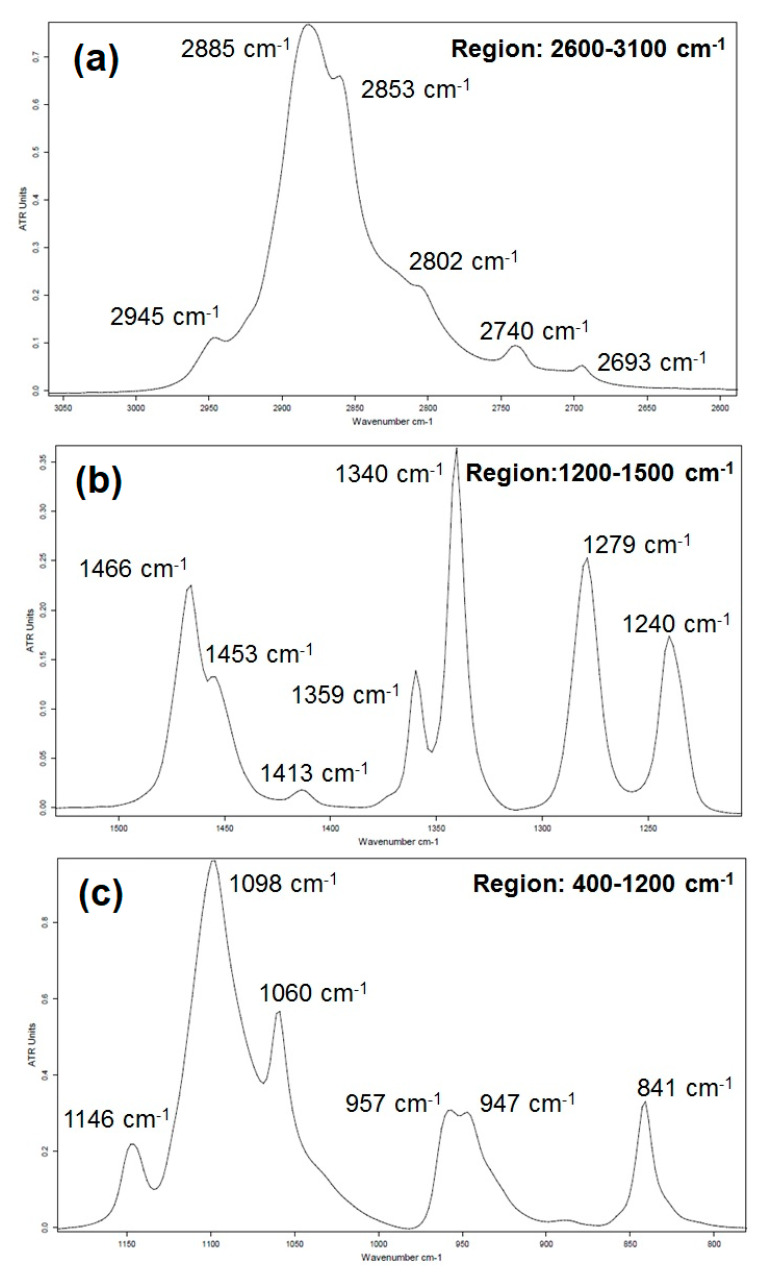
Infrared spectrum (ATR) of PEG in the region (**a**) 3100 to 2600 cm^−1^, (**b**) 1500 to 1200 cm^−1^ and (**c**) 1200 to 800 cm^−1^.

The mid-IR ATR spectrum of PEG is given in Figure 4. Figure 5 represents a focus on the 3100–2600 cm^−1^, 1500–1200 cm^−1^ and 1200–400 cm^−1^ spectral regions. The positions and assignments of the IR absorption bands of the PEG spectrum shown in Figure 4 and Figure 5 will refer to group theory spectral analysis available in the literature studies of PEG [20,21,22,23].

Detailed information about the infrared absorption bands observed in PEG in the spectral regions from 2600 to 3100 cm^−1^, from 1200 to 1500 cm^−1^ and from 800 to 1200 cm^−1^ is reported in Figure 5a, Figure 5b, and Figure 5c, respectively. The IR band assignments of PEG are presented in Table 1.

The PEG’s infrared absorption band assignments are compiled in Table 1. An indication of whether they belong to the crystalline (C) or amorphous (A) phases is reported. Table 1 also gathers each band’s dipole moment orientation with respect to the main chain axis. An oscillating dipole moment parallel and perpendicular to the C-C chain axis is represented by the orientations // and ⊥, respectively. Further analysis of the molecular orientation effects caused by confinement when adsorbed as thin films on Au substrate and chemically modified Au-CH_3_ and Au-NH_2_ substrates will benefit from this information.

The analysis of the crystalline and/or amorphous contributions of the IR vibration bands provides qualitative information about PEG crystallinity in the bulk state. According to reference works [20,21], one can consider the following vibration modes as pure modes related to the crystalline phase, namely:

- the δ_s_(CH_2_) bending mode at 1466 cm^−1^ and the γ_w_(CH_2_) wagging mode doublet at 1340 cm^−1^ and 1359 cm^−1^;
- the triplet of the ν_a_(C-O-C) antisymmetric stretching mode at 1060 cm^−1^, 1098 cm^−1^ and 1146 cm^−1^;
- the γ_r_(CH_2_)_a_ antisymmetric rocking mode at 841 cm^−1^ and the γ_r_(CH_2_) rocking modes of CH_2_ at 957 cm^−1^ and 947 cm^−1^.

All these vibration modes have medium or strong intensities on the IR spectrum allowing them to be used for further determination of conformational analysis and crystallinity changes due to confinement in thin films.

### 3.2. Adsorption of PEG Thin Films on Model Substrates

#### 3.2.1. Surface Topology of Model Substrates

**Figure 6:**
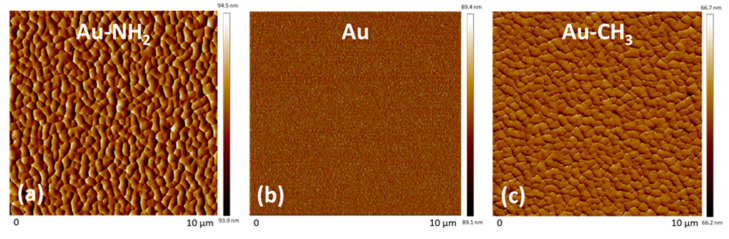
AFM pictures of the deflection signal in contact mode (10 µm × 10 µm) for (**a**) Au-NH_2_ substrate, (**b**) Au substrate and (**c**) Au-CH_3_ substrate.

The surface topology of the Au, Au-NH_2_ and Au-CH_3_ substrates was investigated by AFM in contact mode (Figure 6).

Figure 6b shows that gold deposition on the glass slide allows obtaining a homogeneous gold-coated substrate from the topographic point of view with a very low roughness, i.e., Ra = 0.2 nm. Figure 6a,c show that hexadecanethiol and cysteamine molecules used for the gold-coated substrate functionalization achieve efficient and complete covering of the Au substrate. Moreover, they self-organize into nanodomains of some hundreds of nm. The substrate surface roughnesses are very low, i.e., Ra = 0.7 nm and Ra = 0.6 nm, respectively, for Au-NH_2_ and Au-CH_3_ substrates. These low roughness values will first allow high reflection of the IR wave in PM-IRRAS mode and second, are much lower than the radius of gyration of a single PEG chain, rendering feasible the observation of PEG chain morphology by using AFM.

#### 3.2.2. Adsorption on Gold Substrate (Au): Effect on PEG Chain Organization

**Figure 7:**
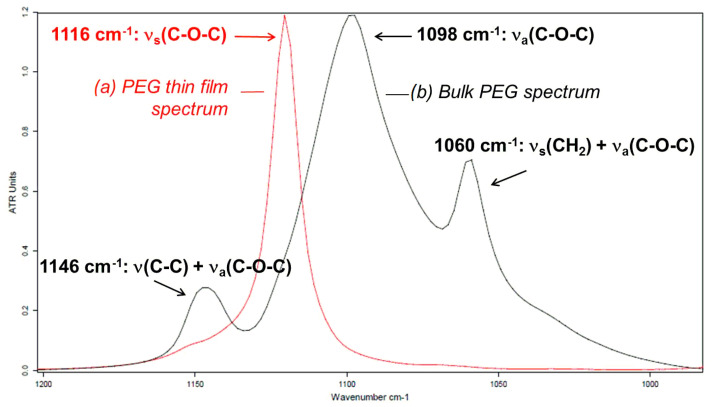
Comparison of the infrared spectra of PEG thin film adsorbed on Au (PM-IRRAS) (red line) and bulk PEG (ATR) (black line) in the ν(C-O-C) region from 1000 to 1200 cm^−1^.

Figure 7 compares the spectrum of bulk PEG obtained by ATR and the spectrum of PEG thin film adsorbed on the gold-coated substrate (referred to as Au) obtained by PM-IRRAS, for the IR spectral region ranging from 1000 cm^−1^ to 1200 cm^−1^ and from 2600 cm^−1^ to 3100 cm^−1^, respectively. The comparison of these spectra is relevant to the effect of the adsorption on the PEG chain orientation. The effect is particularly significant in the region of the ν(C-O-C) stretching vibration modes between 1000 cm^−1^ and 1200 cm^−1^ presented in Figure 7. 

**Figure 8:**
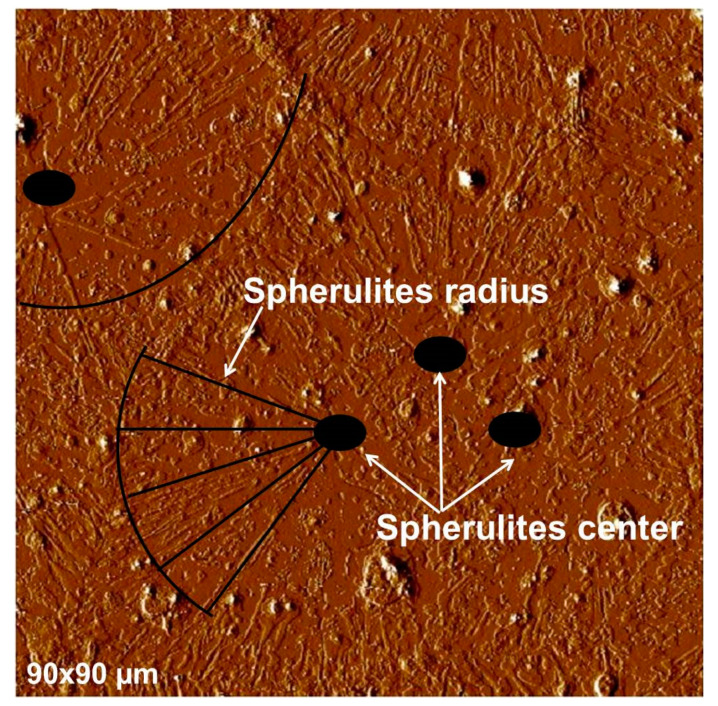
AFM picture of the deflection signal in contact mode for PEG thin film spin-coated on Au substrate (90 µm × 90 µm).

The extinction of the band at 1098 cm^−1^ corresponds to the extinction of the ν_as_(C-O-C) band. The bands at 1060 cm^−1^ and 1146 cm^−1^ are combination bands and their extinction also corresponds to the ν_as_(C-O-C), as their frequencies are located between 1000 cm^−1^ and 1200 cm^−1^, in the region of the C-O-C absorption. The asymmetric stretching mode of C-O-C has a dipole moment parallel to the chain axis and thus parallel to the Au substrate, as this mode is off on the PM-IRRAS spectrum. The parallel orientation of the C-O-C bonds with the Au substrate can be explained by the affinity (partial wetting) of the hydrophilic PEG chains, and especially the oxygen atoms present in C-O-C, with the Au substrate. A single peak emerges at 1116 cm^−1^ on the spectrum corresponding to the symmetric stretching mode ν_s_(C-O-C). The appearance of the ν_s_(C-O-C) symmetric stretching mode with a dipole moment perpendicular to the chain axis supports the hypothesis of the orientation of chains parallel to the Au surface. The extinction of the crystalline peaks at 1060 cm^−1^, 1098 cm^−1^ and 1146 cm^−1^ with the appearance of a single peak emerging at 1116 cm^−1^ is interpreted as an amorphization of PEG [21]. To confirm this amorphization, the surface topography and morphology of the PEG thin film adsorbed on the Au substrate were investigated by atomic force microscopy (AFM) in contact mode (Figure 8).

As seen in Figure 8, a homogeneous layer of PEG covers the gold-coated glass surface indicating PEG chains have an affinity with the Au surface. PEG deposited on Au is mainly arranged radially around a center. The radial orientations of the polymer around a center are attributed to spherulites, crystalline structures characteristic of PEG [24]. In a previous study [25], we have extensively investigated the crystalline behavior of PEG chains using thermal analysis (DSC) and cross-polarized optical microscopy. The PEG melting temperature was found to be equal to 52 °C, and the degree of crystallinity was found to be equal to 95%. Primary and secondary crystallizations were observed by cross-polarized optical microscopy, and spherulite sizes (radius up to 100 µm) and growth rates (up to 20 µm.s^−1^) were recorded as a function of crystallization temperature. This study [25] clearly underlines the crystalline structure in the PEG structure. However, when PEG is spin-coated on gold, the spherulites are only partially formed and not well visible compared to conventional spherulites observed for bulk highly crystalline PEG [25,26,27,28,29]. This observation supports the hypothesis of the PEG amorphization due to polymer adsorption on the Au substrate, as indicated by the extinction of IR crystalline bands on the PM-IRRAS spectrum (Figure 7), and reveals that the PEG/Au interactions are predominant compared to the interchain PEG/PEG interactions.

#### 3.2.3. Influence of the Substrate Chemistry on PEG Chain Organization

**Figure 9:**
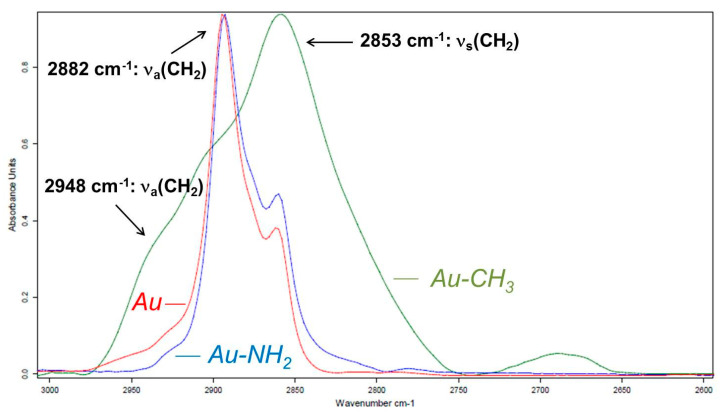
Infrared spectrum of PEG thin film adsorbed on Au substrate (red line), Au-CH_3_ hydrophobic substrate (green line), and Au-NH_2_ hydrophilic substrate (blue line), in the 2600 to 3000 cm^−1^ ν(CH_2_) absorption zone.

Thin films of PEG are deposited on a hydrophilic substrate (Au-NH_2_) and on a hydrophobic substrate (Au-CH_3_). These substrates were developed by chemical grafting of thiols on gold metallic substrates and have different wetting properties, as described in the “Materials” section. The regions of CH_2_ bond stretching modes between 2700 cm^−1^ and 3000 cm^−1^, and C-O-C bond stretching modes ranging from 1000 cm^−1^ to 1200 cm^−1^ being the most sensitive and the most intense, interpretation of the spectra will be carried out for these two spectral zones. Figure 9 compares the PM-IRRAS spectra of PEG thin films deposited on ungrafted Au substrate, on the Au-NH_2_ hydrophilic substrate, and on the Au-CH_3_ hydrophobic substrate, in the ν(CH_2_) stretching vibration mode region.

When the substrate is hydrophobic (Au-CH_3_), a broad absorption band with several contributions appears. Indeed, the absorption band at 2853 cm^−1^ corresponding to the symmetric ν_s_(CH_2_) stretching mode is strongly intensified. The same is true for the asymmetrical (out-of-plane) stretching mode at 2948 cm^−1^, but because this band is not pure (ν_a_(CH_2_) out of plane + ν_a_(CH_3_)), it will not be considered for quantitative analysis. The asymmetric ν_a_(CH_2_) absorption band at 2882 cm^−1^ is reduced in intensity. The increase in the width at the half-height of the absorption bands on the hydrophobic Au-CH_3_ substrate as well as the enhancement of the ν_s_(CH_2_) mode at 2853 cm^−1^ correspond to an amorphization of the PEG.

The hydrophilic substrate (Au-NH_2_) thus seems to be a more favorable surface for the crystallization of PEG than the hydrophobic substrate. However, not all spectral changes of the PEG in a thin film adsorbed on the Au-CH_3_ substrate correspond to the characteristics of amorphization in this region. In addition, the qualitative interpretation of the PM-IRRAS spectra does not allow conclusions to be drawn on the PEG chain orientation relative to the hydrophobic substrate. Quantitative determination of the average angles of inclination of the PEG chains relative to the substrate can then turn out to be essential to access the organization of the PEG chains at these different interfaces.

## 4. Discussion

### 4.1. Quantitative Spectrum Analysis

The quantitative approach consists of deducing, from the PM-IRRAS infrared spectra of adsorbed PEG thin films, the angle of inclination of the macromolecular backbone axis relative to the surface of the substrate. This is accomplished by computing the value of the angle θ, which is defined by the following equation [30] and represents the angle between the dipole moment and the normal to the surface plane.

(1) $$\cos^{2}(\theta )=\frac{I_{\mathrm{anisotropic}}}{3.I_{\mathrm{isotropic}}}$$

**Figure 10:**
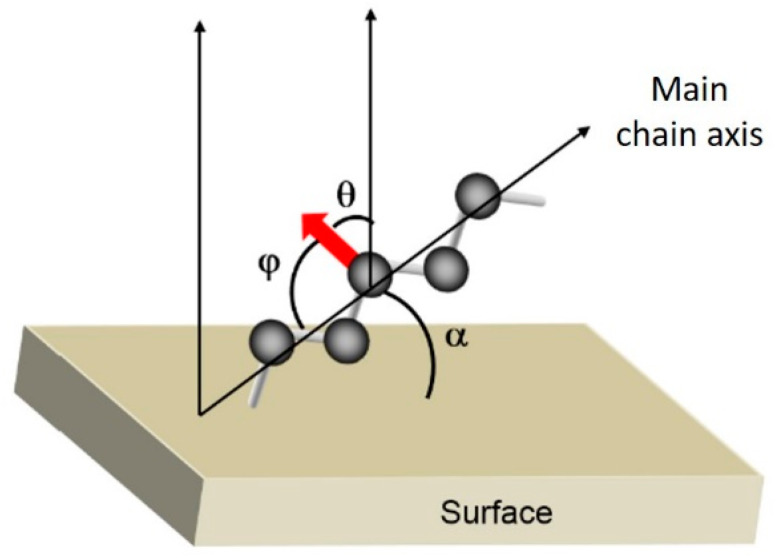
Scheme of orientation angles for a vibration mode with an oscillating dipole (red arrow) ⊥ to the main chain axis. Reproduced from [19], with permission from Elsevier, 2024.

With I_anisotropic_ the intensity measured experimentally for a thin polymer film adsorbed on a substrate (PM-IRRAS) and I_isotropic_ the measured intensity for the same polymer in the bulk state (ATR). In the case of a vibration mode whose transition moment is perpendicular to the chain axis, like the symmetrical elongation mode, the angle φ corresponds to the angle between the dipole moment and the chain axis (see Figure 10), and the angle α corresponds to the angle between the chain backbone axis and the plane of the substrate. Indeed, α is related to φ according to:

α = θ for a vibration mode with an oscillating dipole perpendicular to the chain axis;

α = π/2 − θ for a vibration mode with an oscillating dipole parallel to the chain axis.

The application of PM-IRRAS selection rules and quantitative determination of orientation angles imposes that IR bands satisfy several criteria, namely, to be intense and pure. The orientation of adsorbed polymer chains relative to the substrate, can thus be determined quantitatively by FTIR in PM-IRRAS mode according to Equation (1).

**Figure 11:**
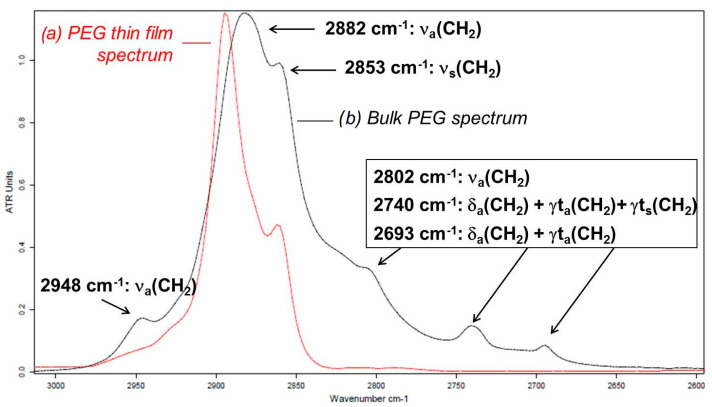
Comparison of the (a) thin film PM-IRRAS and (b) bulk ATR infrared spectra of PEG in the 3100 to 2600 cm^−1^ ν(CH_2_) absorption region.

In the case of PEG, a pure and strong absorption band whose vibration mode corresponds to a dipole moment oriented ⊥ to the chain axis is the ν_s_(CH_2_) absorption band at 2853 cm^−1^. To obtain the inclination of the PEG chains relative to the substrate it is necessary to first carry out a spectral decomposition of the region between 2600 cm^−1^ and 3000 cm^−1^. The positions of the absorption bands are determined by the second derivative treatment of each ATR (bulk) and PM-IRRAS (thin film) PEG spectrum. A comparison of PM-IRRAS and ATR infrared spectra of PEG in the ν(CH_2_) absorption region from 2600 to 3100 cm^−1^ is represented in Figure 11.

**Figure 12:**
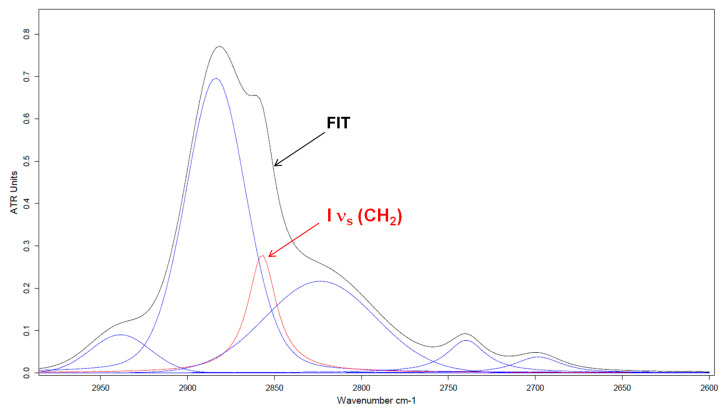
Spectral decomposition of bulk PEG ATR spectrum in the 2600 to 3000 cm^−1^ region. FIT spectrum (black line) corresponds to the sum of individual contributed bands (red line + blue lines).

To perform quantitative analysis, a spectral decomposition where multiple IR bands overlap is needed. To unambiguously identify the precise wavenumbers of characteristic IR vibration bands that overlap, a second derivative mathematical treatment of the initial spectrum was applied. This function improves the resolution of the IR vibration bands, leading to more precise information in terms of band contribution and position. Figure 12 reports an example for the ATR spectrum of PEG in the region of CH_2_ stretching vibration modes. The “FIT” spectrum corresponds to the ATR spectrum obtained by the sum of individual contributed bands (red line + blue lines), resulting from spectral decomposition. 

After spectral decomposition, the area (integrated intensity, I) of the band at 2853 cm^−1^ of the ATR spectrum is compared to the area of the same band of the PM-IRRAS spectrum representative of PEG adsorbed in thin film on different substrates. The area of the absorption band of the different PM-IRRAS spectra requires setting identical values of the position (wavenumber) and the half-height width for the ATR spectrum and the PM-IRRAS spectrum. From these areas and Equation (1), the angle α between the chain axis and the substrate surface is deduced. The values of the areas of the absorption band ν_s_(CH_2_) at 2853 cm^−1^ and orientation angles of the macromolecular chain axis with respect to different substrates are given in Table 2 below.

**Figure 13:**
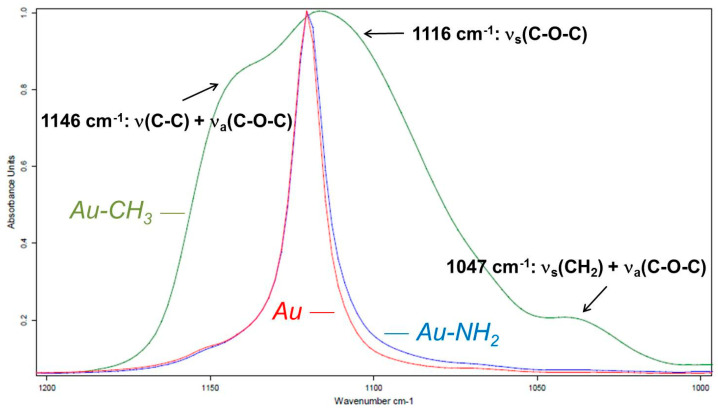
Infrared spectrum of PEG thin film adsorbed on a gold-coated substrate (Au) (red line), hydrophobic substrate (Au-CH_3_) (green line), and hydrophilic substrate (Au-NH_2_) (blue line) in the ν(C-O-C) spectral domain from 1000 to 1200 cm^−1^.

From the values of the angle α, we observe that the inclination of the PEG chains relative to the surface of the substrate is relatively close for the gold substrate (Au) and for the hydrophilic substrate (Au-NH_2_). This trend confirms the hypotheses that the PEG chains are oriented rather // to the surface for both substrates whose contact angles are less than 90° with water. Conversely, the PEG chains tilt significantly on the hydrophobic Au-CH_3_ substrate, reaching an average angle of 62° relative to the plane of the substrate. The fact that PEG chains have a rather hydrophilic character and have little affinity for the hydrophobic surface (hydrophilic/hydrophobic repulsion) could be an explanation. PEG/hydrophobic substrate interactions being disadvantaged, the spectral response of stretching vibration modes of C-O-C bonds between 1000 cm^−1^ and 1200 cm^−1^, which is a region sensitive to PEG crystalline phase, could contribute to understanding the organization of PEG chains. In the C-O-C stretching vibration modes spectral domain, Figure 13 compares the PM-IRRAS spectra of the PEG thin film deposited on the gold substrate (Au) and on both hydrophilic (Au-NH_2_) and hydrophobic (Au-CH_3_) substrates.

The same trend is observed in this region of the infrared spectrum for PEG adsorbed on a hydrophobic substrate: several wide absorption bands replace the narrow absorption bands of more hydrophilic substrates. Indeed, the 1146 cm^−1^ combination band is highly enhanced and an amorphous phase band at 1047 cm^−1^ appears. These two observations are characteristic of the amorphization of PEG, as described previously. This amorphization indicates that the inclined PEG chains relative to the hydrophobic substrate do not organize themselves into lamellae due to their non-stable conformation on this type of substrate. The ν_s_(C-O-C) band at 1116 cm^−1^ corresponds to symmetrical stretching modes with a vibrating dipole ⊥ to the main chain PEG axis. The area of this band calculated from the ATR spectrum can thus be compared to the area of the same band calculated from PM-IRRAS spectra of PEG adsorbed on different substrates. The values of the integrated intensities of the ν_s_(C-O-C) band at 1116 cm^−1^ and the orientation angles of the PEG chains with respect to different substrates are given in Table 3.

The values of the inclination angles of the PEG chains determined from the integrated intensity of the ν_s_(C-O-C) band at 1116 cm^−1^ present the same trend as the values calculated from the symmetrical ν_s_(CH_2_) band at 2853 cm^−1^. Indeed, the inclination of the PEG chains on the Au and Au-NH_2_ substrates is very close. Conversely, the inclination of the PEG chains on the hydrophobic Au-CH_3_ substrate is much more important. Although the inclination angles of the PEG chains follow the same trend, the α values present deviations depending on the absorption band used for quantitative calculations (see Table 2 and Table 3). This discrepancy could come from contributions of the (CH_2_) and (C-C) vibrational modes present in the absorption domain of the (C-O-C) vibrational modes. The broad PM-IRRAS absorption bands of adsorbed PEG on the hydrophobic substrate may suggest that this substrate is less favorable for PEG crystallization. The narrower absorption bands observed on the PM-IRRAS spectrum of PEG adsorbed on the hydrophilic Au-NH_2_ substrate would be characteristic of a more regular arrangement of chains. To ensure the organization of the PEG in thin films on the different model substrates, the widths at the half-height of the absorption bands in the region of ν(C-O-C) are compared in Table 4.

The widths at the half-height of the absorption bands characteristic of vibration modes of the (C-O-C) bonds of PEG are lower for the hydrophilic Au-NH_2_ substrate than for the hydrophobic Au-CH_3_ substrate. These narrow bands may indicate that a structured organization of PEG on these substrates is possible, more particularly on the Au-NH_2_ substrate whose widths at half-height are lower for all the bands in this region of the spectrum. The broad absorption bands of PEG on the Au-CH_3_ substrate indicate a decrease in the crystallinity of PEG on this hydrophobic substrate. A study of the morphology of PEG on these different model substrates would help to support these hypotheses.

### 4.2. Surface Topology of PEG Thin Film

**Figure 14:**
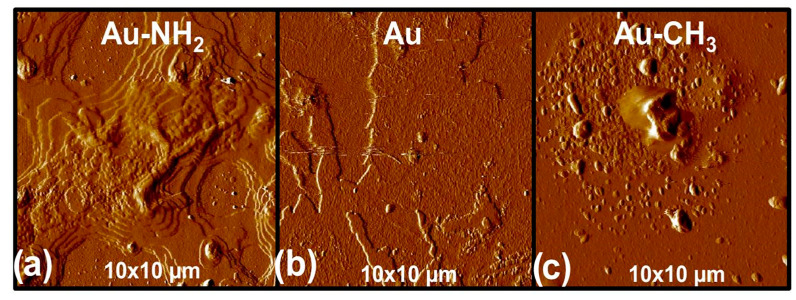
AFM pictures of the deflection signal in contact mode (10 µm × 10 µm) of PEG thin film adsorbed on (**a**) Au-NH_2_ hydrophilic substrate, (**b**) Au gold substrate and (**c**) Au-CH_3_ hydrophobic substrate.

The morphology of PEG thin films on model substrates was characterized by AFM in contact mode. AFM pictures are presented in Figure 14.

On the Au-CH_3_ hydrophobic substrate, the hydrophobic repulsive interfacial forces between PEG and the Au-CH_3_ surface limit the “flat” adsorption of PEG chains. The corresponding AFM picture (Figure 14c) reveals a “dewetting”-like surface pattern of collapsed PEG chains forming chain clusters. As a consequence, the roughness is rather high, i.e., Ra = 60 nm. Thus, the PEG/PEG interchain interactions are favored. The value of the inclination angle (51°) is very close to the magic angle (54°) corresponding to a statistical and isotropic orientation of the transition moments (cos^2^θ = 1/3). Thus, it can be hypothesized that adsorption of the PEG chains on the hydrophobic substrate occurs at the statistical coil state. The surface topology of the PEG deposit on the hydrophobic surface thus explains the value of the inclination angle of the PEG chains inferred from PM-IRRAS infrared spectra and confirms the PEG amorphization. Indeed, the chains being in an unfavorable state cannot organize themselves. On the hydrophilic surface (Figure 14a), the adsorbed PEG thin film adopts a “terraced” surface topology characteristic of a lamellar stacking on the substrate associated with a high roughness value, i.e., Ra = 124 nm. The PEG chains are spread out on the surface, confirming the parallel orientation of the chains relative to the surface area deduced from PM-IRRAS analyses of PEG adsorbed on the Au-NH_2_ surface. The hydrophilic nature of the Au-NH_2_ substrate thus seems to be favorable to the crystallization of PEG chains in the form of stacked “terraces”. Moreover, the surface free energy of the Au-NH_2_ surface is very close to that of PEG, and thus the hydrophilic graft can also explain the ability of PEG chains to crystallize on this substrate. The surface of the hydrophilic Au-NH_2_ substrate is terminated by NH_2_ groups, giving the substrate its hydrophilic character. Lamellar growth parallel to the substrate could then be initiated by the interactions between the PEG chain ends (OH) and NH_2_ groups through hydrogen bonding. One can ask for the consequence of these surface topologies of PEG structures (coil state vs. lamellar stacking) in the wettability of the PEG thin film surface. Nevertheless, determination of the values of water contact angles is not possible on PEG film on Au, PEG film on Au-CH_3_ and PEG film on Au-NH_2_. The reason is that a water droplet swells and solubilizes PEG chains due to the hydrophilic character of PEG. To be significant, contact angles can only be measured with droplets of a non-solvent of PEG chains. One can thus consider contact angles of nonpolar liquids (1-bromonaphtalène (surface tension = 44.4 mJ.m^−2^) or hexadecane (surface tension = 27.5 mJ.m^−2^), but due to their surface tension being lower (hexadecane) or quite equal (1-bromonaphtalène) to the PEG surface tension (43–45 mJ.m^−2^), they fully spread (contact angle ≈ 0°) onto the PEG surface and are irrelevant to support PM-IRRAS or AFM analysis.

**Figure 15:**
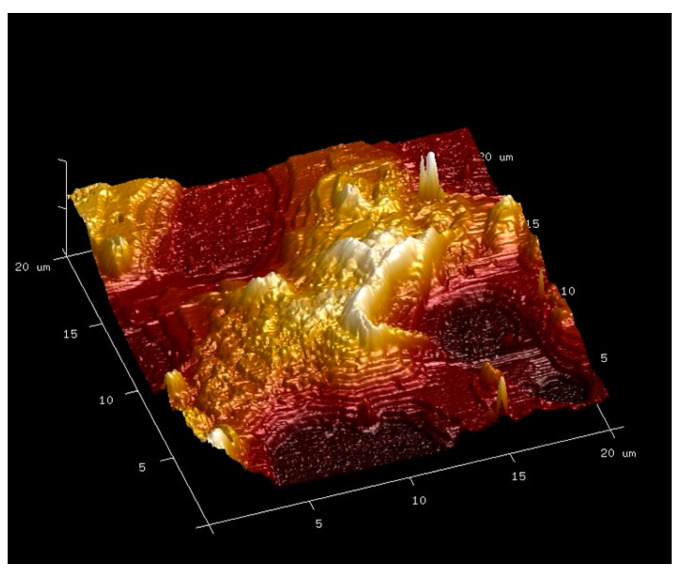
3D AFM picture (20 µm × 20 µm) of the deflection signal in contact mode for PEG thin film spin-coated on the Au-NH_2_ hydrophilic substrate.

A more detailed 3D topographic representation of the PEG thin film on the hydrophilic substrate is given in Figure 15.

The 3-dimensional representation (Figure 15) made it possible to measure the thickness of the terraces equal to approximately 20 nm. According to Yoshihara et al. [20], the length of a PEG chain containing 7 repetitive units is equal to 1.91 nm. The PEG chain with an average molar mass equal to 2050 g.mol^−1^ contains 46 repetitive units and would thus be equal to 12.5 nm. The angle of inclination of the PEG chains relative to the hydrophilic Au-NH_2_ substrate being 25°, the maximum height H reached by the PEG chains is calculated as sin α = H/L, with α the angle of inclination of the PEG chain relative to the Au-NH_2_ substrate, H the maximum height of the chain and L the theoretical length of an extended PEG chain. The maximum height H of a PEG chain on the hydrophilic substrate is then equal to 5.2 nm. The thickness of a terrace step would thus correspond to the stacking of approximately 4 PEG chains. These observations then make it possible to determine adsorption models of PEG chains on these different substrates.

## 5. Conclusions

Previous results showed that PEG adsorbs on substrates having a rather hydrophilic character in a way that the PEG chains spread parallel to the surface. In the case of a very hydrophilic substrate (water contact angle θ = 21°), the adsorbed PEG chains are in a stable thermodynamic state, likewise affinity, which allows them to arrange themselves in an orderly manner (crystallization) after adsorption. Indeed, in the bulk state, PEG crystallizes in the form of very large spherulites [25] of some 100 µm in diameter. On Au-NH_2_ substrates, adsorption of PEG chains is driven by surface chemistry, and PEG crystallizes in the form of stacked lamellas with a thickness equal to 20 nm. On the contrary, on a hydrophobic Au-CH_3_ substrate, the hydrophilic repulsive interfacial forces limit the “flat” adsorption of PEG chains. Thus, the PEG/PEG interchain interactions are favored, and PEG adsorption occurs at the statistical coil state on the hydrophobic substrate. As a consequence, on the hydrophobic Au-CH_3_ substrate, the PEG chains do not crystallize, as the surface energy of the substrate is not favorable to the development of chain/substrate interactions. Surface chemistry, and thus surface energies, significantly influence the adsorption and organization of PEG chains but also influence the crystallization morphology of PEG. Analyses of PM-IRRAS infrared spectra permit qualitative and quantitative approaches to PEG chain adsorption, supported by AFM characterization. These results thus allow the development of models of adsorption of PEG chains.

## Figures and Tables

**Table 1:** IR band assignments for bulk PEG infrared spectrum (ATR).

Wavenumbers (cm^−1^)	Assignment	Phase	Orientation	Intensity
3408	ν(OH)	-	-	Weak-Broad
2945	ν_a_(CH_2_) out of plane + ν_a_(CH_3_)	A; C	-	Weak
2885	ν_a_(CH_2_)	-	//	Strong
2853	ν_s_(CH_2_)	A	⊥	Strong
2802	ν_a_(CH_2_)	-	//	Weak
2740	δ_a_(CH_2_) + γ_t_(CH_2_)_a_ + γ_t_(CH_2_)_s_	-	//	Weak
2693	δ_a_(CH_2_) + γ_t_(CH_2_)_a_	-	⊥	Very weak
1466	δ_s_(CH_2_)	C	⊥	Medium
1453	δ_a_(CH_2_) + δ_s_(CH_2_)	A; C	⊥	Medium
1413	γ_w_(CH_2_)_a_	-	⊥	Weak
1359	γ_w_(CH_2_)_s_	C	⊥	Medium
1340	γ_w_(CH_2_)_a_	C	//	Strong
1279	γ_t_(CH_2_)_a_ + γ_t_(CH_2_)_s_	A; C	⊥	Medium
1240	γ_t_(CH_2_)_a_	C	⊥	Medium
1146	ν(C-C) + ν_a_(C-O-C)	C	//	Strong
1098	ν_a_(C-O-C)	C	//	Very strong
1060	ν_s_(CH_2_) + ν_a_(C-O-C)	C	//	Strong
957	γ_r_(CH_2_)	C	⊥	Medium
947	γ_r_(CH_2_) + ν_a_(C-O-C)	A; C	//	Medium
841	γ_r_(CH_2_)_a_	C	//	Strong

**Table 2:** Band half-widths, band areas, and PEG chain inclination angle α values calculated from the ν_s_(CH_2_) band at 2853 cm^−1^ on substrates of various surface chemistries.

PEG	IR Mode	Width (cm^−1^)	I ν_s_(CH_2_)	α (°)
Bulk	ATR	28	0.386	-
Film on Au		28	0.093	16
Film on Au-NH_2_	PM-IRRAS	28	0.034	10
Film on Au-CH_3_		28	0.900	62

**Table 3:** Band half-widths, band areas and PEG chain inclination angle α values calculated from the symmetrical ν_s_(C-O-C) band at 1116 cm^−1^ on substrates of various surface chemistries.

PEG	IR Mode	Width (cm^−1^)	I ν_s_(CH_2_)	α (°)
Bulk	ATR	29.5	0.946	-
Film on Au		29.5	0.676	29
Film on Au-NH_2_	PM-IRRAS	29.5	0.523	25
Film on Au-CH_3_		29.5	1.732	51

**Table 4:** Half-height widths of PEG absorption bands in the region of C-O-C stretching modes between 1000 cm^−1^ and 1200 cm^−1^.

		Band Half-Height Width	
Wavenumbers	Assignment	Au-NH_2_	Au-CH_3_
1146 cm^−1^	ν_a_(C-O-C) + ν(C-C)	19 cm^−1^	33 cm^−1^
1116 cm^−1^	ν_s_(C-O-C)	12 cm^−1^	55 cm^−1^
1033 cm^−1^	ν_a_(C-O-C) + ν_s_(CH_2_)	-	28 cm^−1^

## Data Availability

The raw data supporting the conclusions of this article will be made available by the authors upon request.
